# Increased Susceptibility to Cortical Spreading Depression in the Mouse Model of Familial Hemiplegic Migraine Type 2

**DOI:** 10.1371/journal.pgen.1002129

**Published:** 2011-06-23

**Authors:** Loredana Leo, Lisa Gherardini, Virginia Barone, Maurizio De Fusco, Daniela Pietrobon, Tommaso Pizzorusso, Giorgio Casari

**Affiliations:** 1 Vita-Salute San Raffaele University and Center for Translational Genomics and Bioinformatics, San Raffaele Scientific Institute, Milan, Italy; 2 Italian Institute of Technology (IIT), Genoa, Italy; 3 CNR Institute of Neuroscience, Pisa, Italy; 4 Department of Biomedical Sciences, University of Padua and CNR Institute of Neuroscience, Padua, Italy; 5 Department of Psychology, University of Florence, Florence, Italy; The Jackson Laboratory, United States of America

## Abstract

Familial hemiplegic migraine type 2 (FHM2) is an autosomal dominant form of migraine with aura that is caused by mutations of the α2-subunit of the Na,K-ATPase, an isoform almost exclusively expressed in astrocytes in the adult brain. We generated the first FHM2 knock-in mouse model carrying the human W887R mutation in the *Atp1a2* orthologous gene. Homozygous *Atp1a2^R887/R887^* mutants died just after birth, while heterozygous *Atp1a2^+/R887^* mice showed no apparent clinical phenotype. The mutant α2 Na,K-ATPase protein was barely detectable in the brain of homozygous mutants and strongly reduced in the brain of heterozygous mutants, likely as a consequence of endoplasmic reticulum retention and subsequent proteasomal degradation, as we demonstrate in transfected cells. *In vivo* analysis of cortical spreading depression (CSD), the phenomenon underlying migraine aura, revealed a decreased induction threshold and an increased velocity of propagation in the heterozygous FHM2 mouse. Since several lines of evidence involve a specific role of the glial α2 Na,K pump in active reuptake of glutamate from the synaptic cleft, we hypothesize that CSD facilitation in the FHM2 mouse model is sustained by inefficient glutamate clearance by astrocytes and consequent increased cortical excitatory neurotransmission. The demonstration that FHM2 and FHM1 mutations share the ability to facilitate induction and propagation of CSD in mouse models further support the role of CSD as a key migraine trigger.

## Author Summary

We previously reported that mutations of the α2 subunit of the Na,K-ATPase cause familial hemiplegic migraine type 2 (FHM2), a dominant form of migraine with aura. This paper describes the first animal model of FHM2 and represents the further proceeding in this disease investigation. Homozygous knock-in mutant mice die just after birth, while heterozygous mice show no apparent clinical phenotype. However, *in vivo* analysis revealed a marked facilitation of cortical spreading depression (CSD), the phenomenon underlying migraine aura. Given the evidence for specific functional coupling between the glial α2 Na,K pump and glutamate transporters, we hypothesize that CSD facilitation in the FHM2 mouse model is sustained by inefficient glutamate clearance by astrocytes and consequent increased cortical excitatory neurotransmission. We finally propose this FHM2 mouse as a valuable *in vivo* model to investigate migraine mechanisms and, possibly, treatments.

## Introduction

Migraine is a clinically heterogeneous disorder affecting more than 10% of the general population. It generally occurs with unilateral and pulsating severe headache often accompanied by nausea, photophobia and phonophobia. In approximately one third of migraineurs, the headache attack is preceded by aura, a transient neurological symptom that are most frequently visual but may involve other senses [1]. The migraine attack is triggered by a brain dysfunction that leads to activation and sensitization of the trigeminovascular system, particularly trigeminal nociceptive afferents innervating the meninges and lastly to headache [2], [3], [4]. Neuroimaging examination suggests that migraine aura is associated to cortical spreading depression (CSD), a short-lasting, intense wave of neuronal and glial cell depolarization. CSD spreads slowly over the cortex at a rate of approximately 2–5 mm/min and is followed by long lasting depression of neuronal activity [5], [6], [7], [8]. Experimental evidence on patients and animal models supports CSD as both underlying migraine aura [1], [7], [8], [9] and a key triggering event for trigeminal activation [10], [11], [12], although the role of CSD in migraine headache is still debated. As an indirect confirmation, several migraine prophylactic agents cause an increase of CSD initiation threshold [13].

Common migraine has a strong multifactorial genetic component, which is higher in migraine with aura (MA) than in migraine without aura (MO) [14], [15]. As for many other multifactorial diseases whose complexity hampers the investigation of the pathogenetic mechanisms, rare monogenic forms that phenocopy most or all the clinical features of the common disease are of great help for describing the complicated events leading to migraine. Familial hemiplegic migraine (FHM) is a rare autosomal dominant subtype of MA, whose aura symptoms include hemiparesis. Aura symptoms and headache duration are usually longer in FHM than MA, but all other headache properties are similar. FHM is genetically heterogeneous and is associated to mutations in three different genes. Mutations in *CACNA1A* [16], *ATP1A2* [17] and *SCN1A* [18] genes are responsible for Familial hemiplegic migraine type 1 (FHM1), type 2 (FHM2), and type 3 (FHM3), respectively. The *CACNA1A* and *SCN1A* genes both encode neuronal voltage-gated ion channels, whereas the *ATP1A2* gene encodes the α2 subunit of the Na,K-ATPase, hence suggesting a key role of cation trafficking in the pathophysiology of FHM.

Until now, more than 50 FHM2 mutations have been identified and most of these are missense mutations. A small fraction of mutations is represented by microdeletions [19] and a single mutation affecting the stop codon, which causes an extension of the ATP1A2 protein by 27 aminoacid residues [20]. Most of the *ATP1A2* mutations are associated with pure FHM without additional clinical symptoms [17], [19], [20], [21], [22]. However, a number of FHM2 mutations have been associated to complications like cerebellar ataxia [23], childhood convulsions [24], epilepsy [25] and mental retardation [26]. Interestingly, ATP1A2 mutations associated with non-hemiplegic migraine phenotypes, such as basilar migraine and even common migraine have been reported [27], [28].

The Na,K ATPase is a P-type ion pump that utilizes the free energy of ATP hydrolysis to exchange Na^+^ for K^+^ and maintains gross cellular homeostasis. The functional pump is a heterodimer, consisting of one α catalytic subunit and one β subunit that is required for protein folding, assembling, membrane-addressing, and modulates substrate affinity [29]. The α subunit exposes both the amino- and carboxy- termini in the cytoplasm and crosses the plasma membrane with ten transmembrane segments (M1–M10) [30]. Four isoforms of α Na,K-ATPase (α1, α2, α3 and α4) are present in mammals [29], [31]. While no pathogenic mutations are known for the ubiquitous α1- and the testis α4-subunits, mutation in both α2 and α3 isoforms cause neurological diseases when mutated, FHM2 and rapid-onset dystonia parkinsonism, respectively [32]. While in the adult brain the α1 isoform is nonspecifically present in both neurons and glial cells and α3 is neuron-specific, the α2 isoform is essentially expressed in astrocytes [33].

Investigation of the functional consequences of FHM2 mutations in heterologous expression systems revealed that these mutations produce partial or complete loss of function of the α2 Na,K pump [34], [35], [36]. Here, we report the generation of the first mouse model of FHM type 2, a knock-in mutant harboring the W887R ATP1A2 mutation.

The W887R mutation localizes to the extracellular loop between M7 and M8, which includes the β subunit binding site [37] and was shown to produce the almost complete loss of pump activity [17], [38]. Homozygous *Atp1a2^R887/R887^* mutants die just after birth, while heterozygous *Atp1a2^+/R887^* mice are fertile and show no apparent clinical phenotype. However, heterozygous FHM2 mouse displays altered CSD properties, such as decreased threshold and increased velocity of propagation. We hypothesize that inefficient astrocyte-mediated clearance of glutamate from the synaptic cleft is a key event for the enhanced susceptibility to CSD in the FHM2 mouse.

## Results

### Generation of FHM2 knock-in mutant mouse

**Figure 1:**
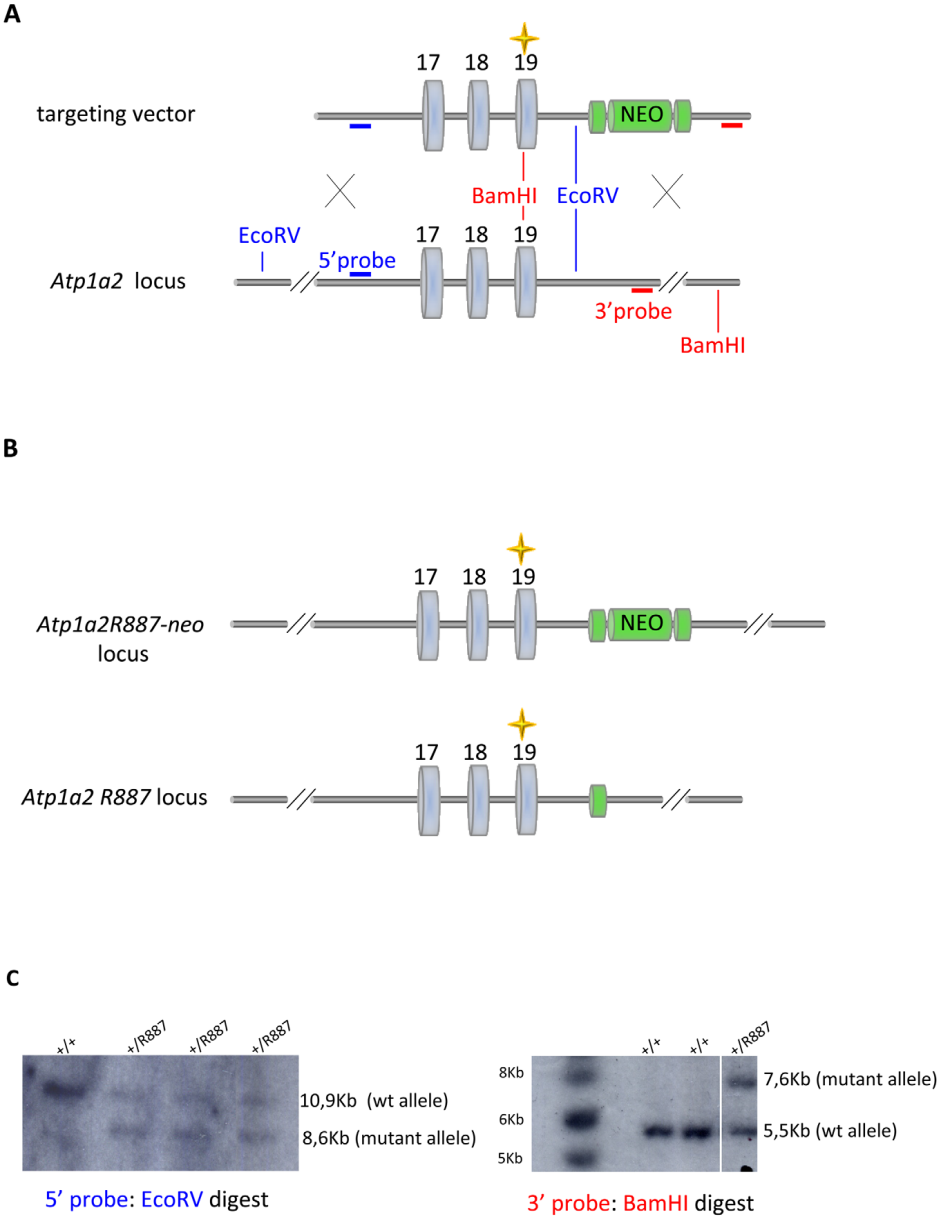
Knock-in construct strategy. A. Genomic structure of the targeting vector and wt *Atp1a2* allele. B. Predicted structure after homologous recombination (*Atp1a2^R887-neo^* allele), and after Flp-mediated deletion of the neo-cassette (*Atp1a2^R887^* allele). FRT sites are indicated by green circles. Grey boxes indicate respective exons, and yellow star the R887 mutation in exon19. C. Southern blot of F1 *Atp1a2^+/R887^* ^-neo^ mutant mice. EcoRV and BamHI-digested genomic DNA from two genotypes for wild-type and *Atp1a2^+/R887neo^* mutant strains probed 5′ and 3′ probe.

With the aim of investigating the molecular pathogenesis of FHM type 2, we generated a knock-in mouse model by inserting an FHM2 mutation, the transition T2763C that causes the aminoacid replacement W887R in the *Atp1a2* murine gene (construct details in M&M). The amino acid sequence conservation between human and mouse α2 Na,K-ATPase proteins is very high and, in particular, in the extracellular domain between transmembrane domains M7–M8, where W887R is located [17]. This mutation was one of the first two mutations reported to be associated to typical cases of the disease. Embryonic stem cells harboring the R887 and the *neo* cassette were injected in C57Bl/6J blastocysts and then transferred to pseudopregnant CD1 females. We obtained three chimeric mice, one of which transmitted the *Atp1a2^+/R887-neo^* allele through germline (Figure 1A). Heterozygous *Atp1a2^+/R887-neo^* mice were genotyped by Southern blot analysis (Figure 1C), are fertile and display no apparent phenotype. To remove the neo cassette that hampers the natural expression of the mutant allele, we crossed the *Atp1a2^+/R887-neo^* mice with transgenic mice expressing the Flippase recombination enzyme (FLPe) under the control of the human ACTB promoter (*TgN(ACTFLPe)9205Dym; The Jackson Laboratory*). Hence, we obtained the heterozygous *Atp1a2^+/R887^* knock-in mice (Figure 1B), which are fertile as well and show no visible clinical phenotype. Contrary to heterozygous mice, homozygous *Atp1a2^R887/R887^* mutants do not survive beyond the first day *post partum*, thus resembling the neonatal lethal phenotype of the *Atp1a2* null mutant [39], which succumbs for dysfunctional neuronal activity and respiratory distress. Therefore, we addressed our investigation onto the heterozygous knock-in mouse, which shares the *Atp1a2* gene asset with FHM2 patient. The general behavior of heterozygous *Atp1a2* ^+/R887^ mice was tested by a modified SHIRPA protocol [40] that provides comparable quantitative data on animal motor, sensory, autonomic and neuropsychiatric functions. The scored parameters are summarized in Table 1. No major differences in the sensory-motor functions were observed between heterozygous *Atp1a2^+/R887^* (n = 8) and wild-type (n = 6) mice, except for a higher fear and anxiety of *Atp1a2^+/R887^* at the specific tests of transfer arousal and fear (p<0.05; Table 2).

**Table 1:** Parameters scored in SHIRPA primary screen.

	Parameter	Score	Range of scores
Viewing Jar	Body Position	0-5	Completely flat to repeated leaping
	Spontaneous Activity	0-3	None to rapid movement
	Respiration Rate	0-3	Gasping to hyperventilation
	Tremors	0-2	None to marked
Arena	Transfer Arousal	0-6	Coma to extremely excited
	Palpebral Closing	0-2	Eyes wide open to closed
	Piloerection	0-1	Absent or present
	Gait	0-4	Normal to incapacity
	Pelvic Elevation	0-3	Markedly flattened to elevated
	Tail Elevation	0-4	Dragging to Straub
	Touch Escape	0-3	None to extremely vigorous
	Positional Passivity	0-4	Struggles or not when held by tail
	Trunk curl	0-1	Absent or present
	Limb Grasping	0-1	Absent or present
Tail Suspension	Grip Strength	0-4	None to unusually effective
	Body Tone	0-2	Flaccid to extreme resistance
	Toe Pinch	0-4	No withdrawal to very brisk
Supine Restraint	Heart Rate	0-2	Bradycardia to tachycardia
	Skin Color	0-3	Pale to Pigmented
	Limb Tone	0-4	No resistance to extreme resistance
	Abdominal Tone	0-2	Flaccid to extreme resistance
	Lacrimation	0-3	Normal eye to excessive lacrimation
	Salivation	0-2	None to extreme wetness
	Provoked Biting	0-1	Absent or present
Test Arena	Righting Reflex (flip)	0-3	Landing on feet to failing to right
	Negative Geotaxis	0-3	Climbing the grid to no movement
	Vocalization	0-1	Absent or present
	Irritability	0-1	Absent or present
	Aggression	0-1	Absent or present
	Fear	0-1	Absent or present

**Table 2:** SHIRPA results.

Tests	Wild type	*Atp1a2^+/R887^*
Spontaneous activity	1.67±0.82	1.87±0.64
Transfer arousal*	3.00±0.76	4.67±0.52
Tail elevation	1.38±0.52	1.00±0.00
Touch escape	2.25±0.71	2.33±0.82
Grip strength	1.63±0.52	2.00±0.00
Trunk curl	1.00±0.00	1.34±0.52
Heart rate	1.88±0.35	1.34±0.52
Vocalization	0.63±0.52	1.00±0.00
Irritability	0.63±0.52	0.67±0.52
Aggression	0.38±0.52	0.34±0.52
Fear*	1.00±0.00	0.17±0.41

SHIRPA primary screening was used to assess sensory-and motor function of *Atp1a2^+/R887^* knock-in mice (n = 8) lines. Wild-type littermates (n = 6) were used as control. All data are expressed as mean ± S.D.. Significant Mann-Whitney non-parametric test is indicated (* = p<0.05).

### In vivo expression of mutant α2- Na,K-ATPase

**Figure 2:**
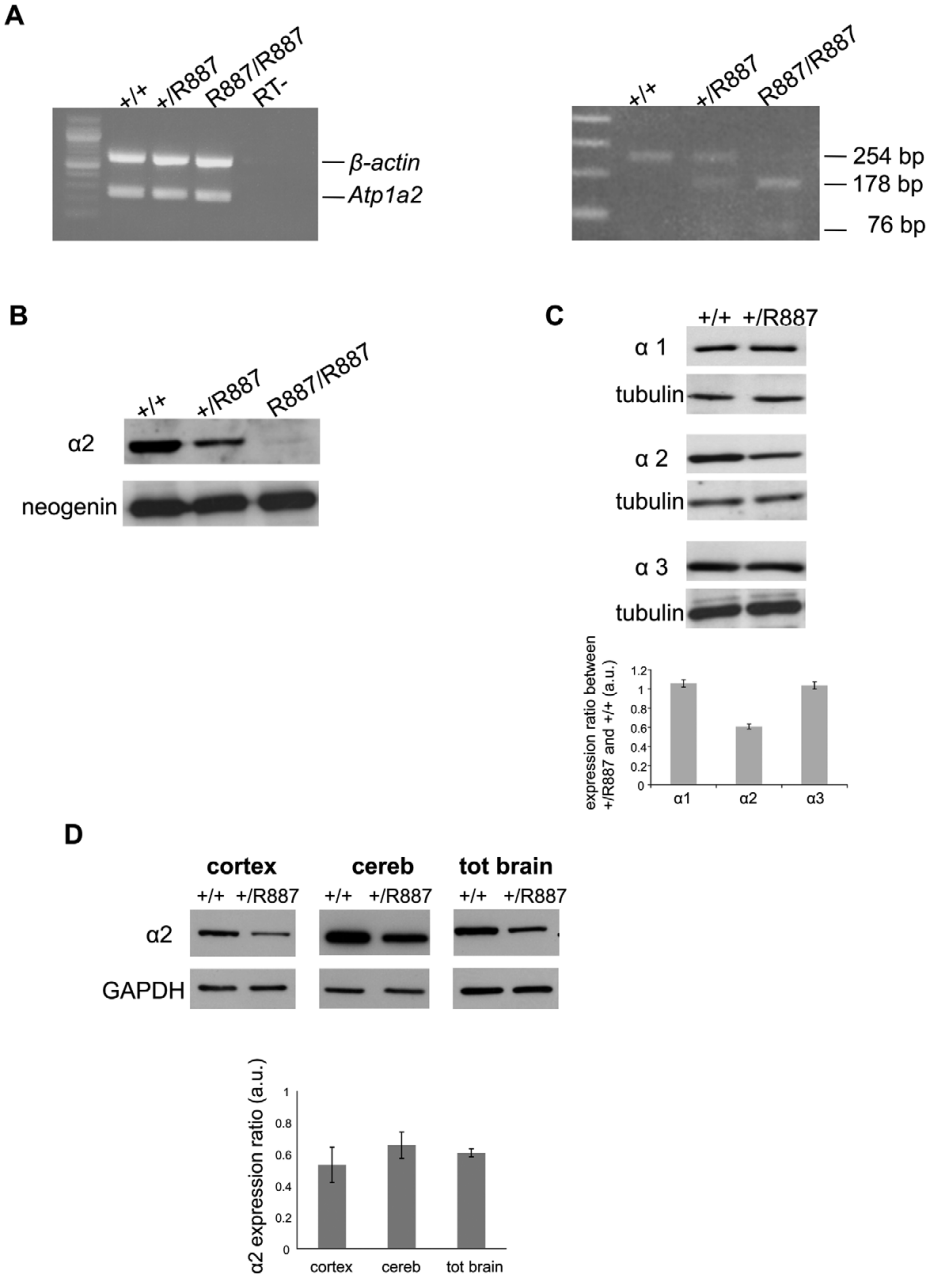
In vivo expression of mutant *Atp1a2*. Total RNA and protein samples were isolated from brain of wild type (+/+), *Atp1a2^+/R887^* (+/R887) and homozygous *Atp1a2^R887/R887^* (R887/R887) mice at E19.5. A. Left panel. Semi quantitative *Atp1a2* RT-PCR (254 bp fragment) on brain cDNA. The b-actin fragment (610 bp) is included as in-tube normalizer. Right panel, same *Atp1a2* RT-PCR fragments digested by MspI. B. Protein blot of microsomal fraction probed with anti-α2 Na,K-ATPase antibody and anti-neogenin as loading control; the α2 Na,K-ATPase and neogenin bands appear at the expected size of 110 kDa and 52 kDa, respectively. C. Total brain lysates from adult wild type and *Atp1a2^+/R887^* mice probed with anti-α1, α2, and α3 Na,K-ATPase antibodies; anti- tubulin as loading control. Densitometric quantization shows a 50% reduction of the heterozygous mutant α2 level compared to wild type. Error bars represent ± SD; Student's *t test* p<0.05, n = 3; α1, α2, and α3 Na,K-ATPases migrate as single bands according to the expected size of 110 kDa. D. Region specific immunoblot from cortex, cerebellum and total brain of adult wild type and *Atp1a2^+/R887^* mice probed with anti-α2 and anti- glyceraldehyde-3-phosphate dehydrogenase (GAPDH) as loading control (Figure 2D). Densitometry evaluation shows significant reduction in α2 level (p<0.05) of 50%, 35% and 40% in cortex, cerebellum and total brain, respectively. Error bars represent ± SD; Student's *t test* p<0.05, n = 3.

Mutant and wild type *Atp1a2* gene expression was evaluated at E19.5 in *Atp1a2^R887/R887^* for lethality constrain and at adult age in *Atp1a2^+/R887^* mutants. Semi quantitative reverse transcription PCR (RT-PCR) analysis showed that both wild type and mutant *Atp1a2^R887^* alleles express equal amount of transcripts (Figure 2A, left panel). Each RT-PCR experiment was normalized on intra-sample actin transcript level. The nucleotide replacement resulting in the W887R mutation creates a new MspI restriction site that we used to confirm the R887 mutation in the *Atp1a2^R887^* transcript and to quantify the mutant transcript in heterozygous mice as intra-sample control (Figure 2A, right panel). The expression of α2 Na,K-ATPase protein was assessed in embryonic brain. Immunoblot of total lysate and microsomal fractions revealed a markedly reduced amount of α2 Na,K-ATPase in the mutants, which displayed approximately half the level of wild type in the *Atp1a2* ^+/R887^ mice. In the *Atp1a2^R887/R887^* mutant, the R887 α2 Na,K-ATPase is barely observable (Figure 2B). In order to investigate whether the reduced amount of α2 isoform induces a compensatory increase of expression of the paralogous α1 and α3 isoforms in the adult, when the principal phenotype, CSD, is assessable, we analyzed brain tissues with specific α isoform antibodies. No differences in the level of α1 and α3 isoforms were observed in whole brain of *Atp1a2* ^+/R887^ mice (Figure 2C) compared to wild type ones. On the contrary, the *Atp1a2* ^+/R887^ model displays an α2 expression level reduced to approximately 50% in the cortex, 35% in cerebellum and 40% in total brain (Figure 2D).

### Mutant R887 alpha 2 ATPase is poorly exported to the plasmamembrane

**Figure 3:**
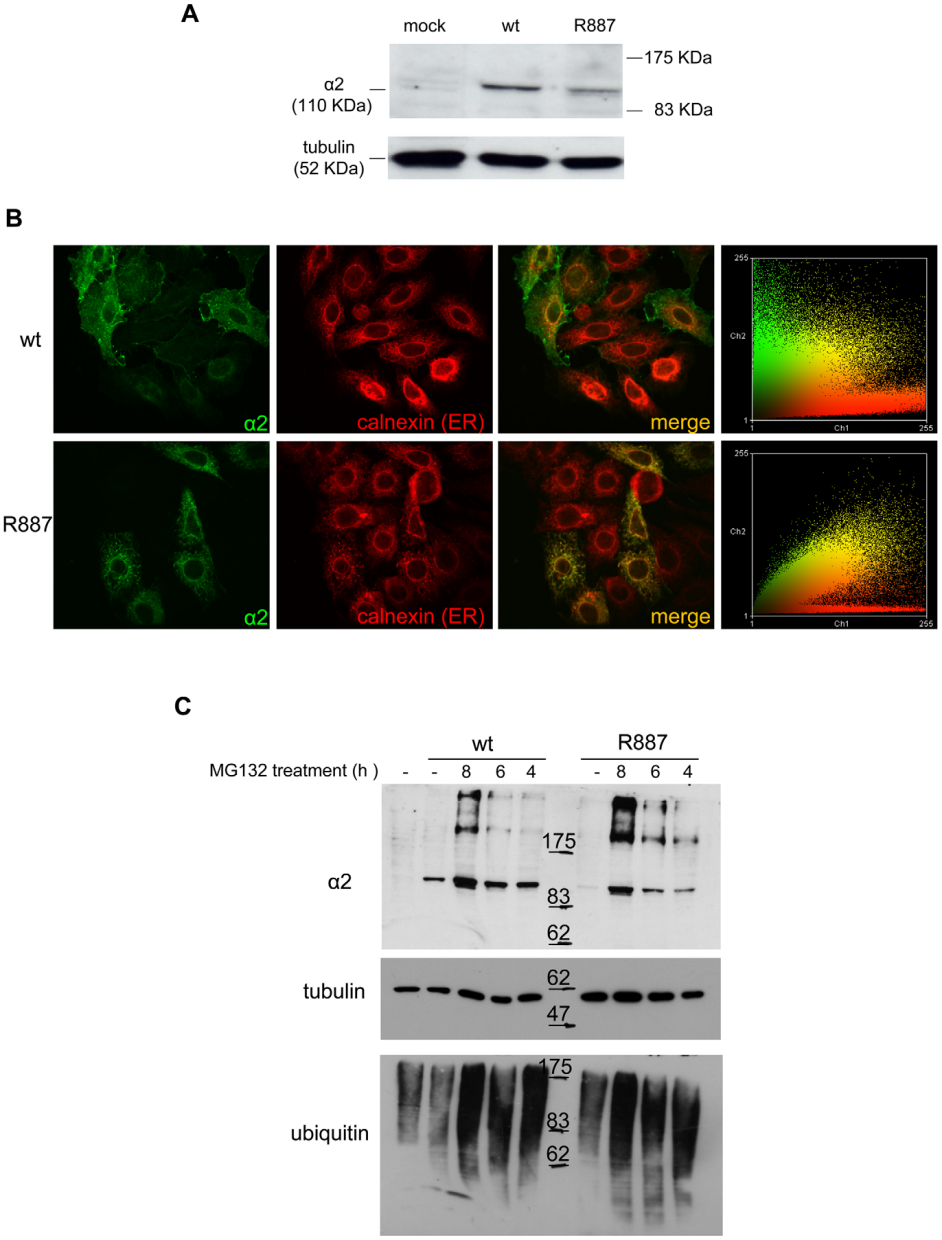
In vitro expression and localization of *α2-ATPase*. A. Immunoblot of HeLa cells transfected with pA2-wt-Myc (wt) and pA2-R887-Myc (R887) probed with anti α2-ATPase antibody. B. Calnexin (red) and α2 ATPase (probed with anti-myc; green) immunofluorescence staining of wt or R887 transfected Hela cells. R887 α2 ATPase mostly colocalises with the endoplasmic reticulum. In the right graphs, the scatter plot of red (ch1) vs. green (ch2) colocalization intensities. (wt parameters: Rr 0.369, R 0.6, Ch1:Ch2 0.999; R887 parameters: Rr 0.558, R 0.755, Ch1:Ch2 0.997). C. HeLa cells expressing wt or R887 variant of α2 ATPase are treated with the reversible proteasome inhibitor MG132 (10 µM for 4, 6 and 8 hrs). Blots are probed with anti- α2 isoform antibody, anti-ubiquitin antibody and anti-tubulin as loading control.

The loss of α2 protein in the *Atp1a2^R887/R887^* prompted us to investigate the fate of wild-type and mutant α2 proteins by cell transfections. HeLa cells were co-transfected with pA2-R887 or pA2-wt constructs, which express, respectively, mutant and wild type full length c-myc-tagged *ATP1A2* cDNAs, each together with pB2 expressing the β2 subunit (as described in [17]). Immunoblot revealed a decreased amount of R887 mutant (Figure 3A), thus confirming the *in vivo* results on *Atp1a2^R887/R887^* and *Atp1a2^+/R887^* mutant mice. More important, immunofluorescence staining demonstrated a different subcellular localization. Wild type α2 Na,K ATPase showed a typical plasmamembrane and slightly endoplasmic reticulum staining (Figure 3B, upper panels). Differently, most of R887 mutant protein appeared as punctuated pattern localized in the perinuclear region, which overlapped with the endoplasmic reticulum marker calnexin (Figure 3B; a colocalization quantification appears on right panels). Misfolding of the mutant α2 Na,K ATPase induced by the R887 mutation causes very likely the endoplasmic reticulum retention and the inefficient and delayed secretion process. In fact, by inhibiting the proteasome activity with MG132, wild-type and more consistently mutant α2 subunits accumulated in transfected cells (Figure 3C).

### *Atp1a2^+/R887^* knock-in mice are more prone to cortical spreading depression

**Figure 4:**
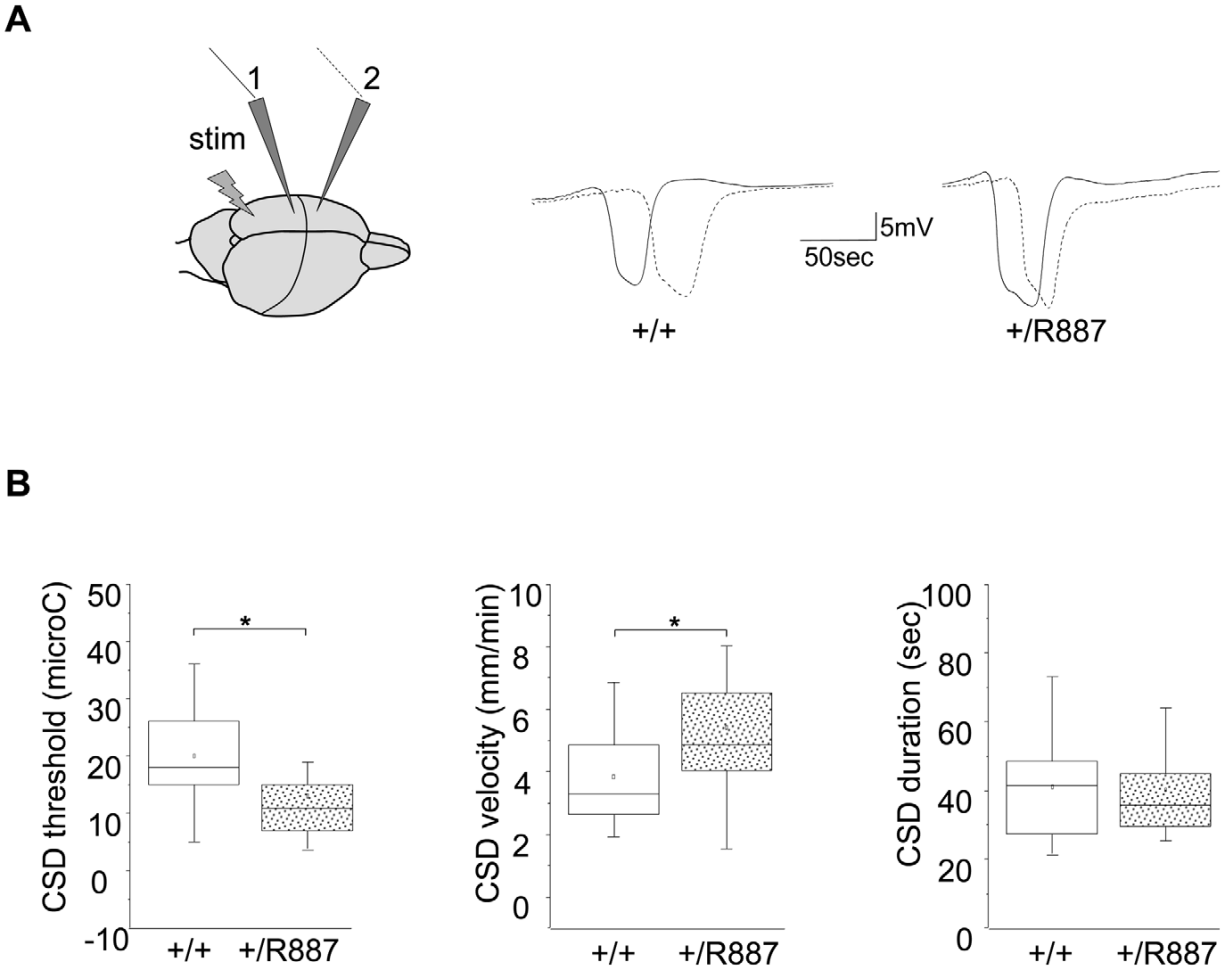
In vivo CSD recording. A. On the left, a sketch of the positioning of the stimulating (stim) and recording electrodes (1, 2) is outlined. Example of traces recorded in wild type (+/+) and *Atp1a2* ^+/R887^ mutants (+/R887) at the two electrodes with dotted line indicating the wave recorded at the first electrode and full line indicating the wave recorded at the second electrode in both wild type and mutated animals. B. CSD threshold, velocity and duration of wild type and mutant are reported in box plots reporting bottom-up the 5^th^ percentile, 25^th^ percentile, median, 75^th^ percentile and 95^th^ percentile. The single dot represents the mean value described for each group. *Atp1a2* ^+/R887^ heterozygous animals are more sensitive to CSD induction (+/R887, 13.00±1.7 µC, n = 20; +/+, 19.9±1.9 µC, n = 18; p<0.01; left graph). CSD velocity of propagation is increased in *Atp1a2* ^+/R887^ mutants (wild type, 3.85±0.35 mm/min n = 18); mutant, 5.41±0.41 mm/min, n = 20; p<0.01; middle graph). No significant difference was observed in CSD duration (wild type 41.1±3.5 sec, n = 18; *Atp1a2* ^+/R887^ 40.1±3.19 sec, n = 20; p = 0.83; right graph).

Migraine is a complex phenotype that hampers the objective and quantitative evaluation in animal models. In order to assess the effect of the R887 *ATP1A2* mutation on an important component of the migraine attack, cortical spreading depression (CSD), we analyzed this neuronal phenomenon in adult *Atp1a2* ^+/R887^ mice and in their wild type littermates. CSD was induced by electrical stimulation of the visual cortex using a bipolar electrode and recorded at two sites of somatosensory and motor cortex (Figure 4A). Incremental current stimuli were delivered up to CSD induction and the charge delivered at CSD activation was considered as threshold.

*Atp1a2* ^+/R887^mutants were more susceptible to undergo CSD. Indeed the threshold for induction of CSD in mutant animals was significantly lower than wild type animals (*Atp1a2* ^+/R887^, 13.00±1.7 µC, n = 20; wild type, 19.9±1.9 µC, n = 18; t-test p<0.01) (Figure 4B, left graph). Moreover, CSD propagation rate was altered in the mutant, which showed higher CSD velocity rising from 3.85±0.35 mm/min (wild type, n = 18) to 5.41±0.41 mm/min (n = 20; t-test p<0.01) in *Atp1a2* ^+/R887^ mice (Figure 4B, middle graph). No significant difference was observed in CSD duration (*Atp1a2* ^+/R887^ 40.1±3.19 sec, n = 20; wild type 41.1±3.5 sec, n = 18; t-test p = 0.83) (Figure 4B, right graph). After the first CSD, the trace was monitored for further 90 minutes to reveal repetitive CSDs, a parameter correlated with the phenotype severity of FHM models [41]. Heterozygous R887 mutation did not modify the proportion of mice showing repetitive CSD (*Atp1a2* ^+/R887^ 4 out of 20 mice, wild type 4 out of 18 mice). We conclude that the R887 α2 Na,K-ATPase facilitates CSD induction and propagation, but it neither affects its duration nor promotes the induction of repetitive CSDs. Within groups, no difference in CSD threshold and propagation rate was observed between male and female (see Methods).

## Discussion

Genetic mouse models are essential tools to dissect complex pathogenic mechanisms leading to human diseases. Here, we report data on the generation of the first knock-in mouse carrying the W887R ATP1A2 mutation causing FHM2. The R887 allele has been associated to a typical form of FHM with hemiparesis and epileptic episodes [17]. Since the effect of this mutation is an almost complete loss of function [17], [38], we expected a neonatal lethality of homozygous mutant mice. In fact, *Atp1a2^R887/R887^* mutants die few minutes after birth, closely resembling the knock-out models [39], [42], which fail to develop a regular respiratory rhythm [43], [44]. Heterozygous *Atp1a2* ^+/R887^ mice are viable and fertile. A general behavior characterization by a modified SHIRPA protocol [40] shows a higher susceptibility to fear and anxiety in *Atp1a2^+/R887^* mice. This result resembles the previous reports by Ikeda et al. [42] and Moseley et al [45] showing similar phenotype in the null allele heterozygous Atp1a2^+/−^ mice by more specific tests for anxiety and conditioned fear.

As we demonstrate, the mutant gene is correctly transcribed and translated. However, the mutant R887 protein is ineffectively exported from the endoplasmic reticulum-Golgi system. Mutant R887 isoform is mostly degraded by the proteasomal system as demonstrated by the remarkable accumulation of mutant protein under proteasomal inhibition. This is particularly evident *in vivo*, where the mutant protein is barely detectable in the homozygous mutant brain. This finding is apparently in contrast with our previous result [17] that showed the mutant R887 subunit localized all over the cytoplasm in COS7 transfected cells and seemingly to plasmamembrane as well. By the recent confocal analysis and employing a transfection system that does not saturate, like the COS7 cells, the cytoplasm of exogenous protein, the mutant protein is shown as mostly endoplasmic reticulum-retained.

It is worth noting that Koenderink and coworkers proposed a plasmamembrane localization of the R887 protein by centrifugal fractionation in Xenopus oocytes, probably due to the different cellular system and conditions (room temperature) and the indirect method of localization [38]. Infact, this test at room temperature may favor the mislocalization of the α2 ATPase mutant protein, as reported in [36]. Considering the autosomal dominant inheritance of FHM, we have addressed our attention to the phenotype analysis of the heterozygous knock-in mouse. CSD represents an excellent phenotype to be analyzed in animal models of migraine as CSD underlies migraine aura in patients [1], [7], [8], [9] and can activate the meningeal trigeminal nociceptors in animals [12]. *Atp1a2^+/R887^* mutant mice, our FHM2 model, are more susceptible to CSD as shown by the decreased threshold of induction and the increased velocity of propagation of CSD induced by electrical stimulation of the cortex in vivo. Duration of CSD in *Atp1a2^+/R887^* mice is unchanged. The facilitation of CSD in our FHM2 mouse model is thus very similar to that previously described in *Cacna1a* knock-in mice representing the FHM1 models [41], [46]. In fact, both homozygous and heterozygous S218L and homozygous R192Q FHM1 models showed a lower threshold for CSD induction and a higher velocity of CSD propagation, whereas CSD duration was not significantly prolonged. Interestingly, the extent of CSD facilitation correlated with the severity of the clinical phenotype of the two FHM1 mutations in humans [41], [46], [47]. The demonstration that FHM2 and FHM1 mutations share the ability to facilitate induction and propagation of CSD in mouse models further support the role of CSD as a key migraine trigger.

The facilitation of CSD in *Atp1a2^+/R887^* mice could be due to impaired clearance of K^+^ and/or glutamate by astrocytes during cortical neuronal activity consequent to loss-of-function of the α2 Na,K ATPase pump, as previously suggested [34], [48]. Pharmacological evidence shows that α3 and/or α2 Na,K pumps participate in the clearance of K^+^ from the extracellular space during intense neuronal activity, although the relative importance of α3 and α2 Na,K pumps is unclear[49], [50]. Most models of CSD include local increase of extracellular [K^+^] above a critical value as a triggering event in the initiation of CSD, hence predicting that a reduced K^+^ clearance would result in a lower threshold for CSD induction [51]. Indeed, in hippocampal slices the inhibition of α2 and α3 Na,K pumps by local administration of ouabain (at a concentration which only partially affects the low affinity α1 Na,K pump) reduced the threshold for CSD induction by local pulses of high [K^+^] [52]. This reduced CSD threshold was accompanied by a large increase in CSD duration (and decrease in post-CSD undershoots of membrane potential and external [K^+^]), pointing to the involvement of α3 and/or α2 Na,K pump activity in CSD termination. We speculate that our findings of a lower threshold for CSD induction but unaltered CSD duration in *Atp1a2^+/R887^* mice suggest a relatively minor role of the glial α2 Na,K pump in K^+^ clearance. This is in agreement with the evidence that the α3 isoform contributes most of the Na,K ATPase activity in mouse brain homogenates [53] and, therefore, we suggest that the reduced CSD threshold in FHM2 knockin mice is not primarily due to impaired K^+^ clearance by astrocytes.

Several lines of evidence indicate a specific role of the α2 Na,K pump in glutamate clearance during synaptic transmission. The α2 Na,K pump is specifically stimulated by glutamate in cultured astrocytes [54]. In the adult somatosensory cortex the α2 Na,K pump shows a specific localization in astrocyte processes surrounding glutamatergic synaptic junctions, which coincides with that of the glial glutamate transporters GLAST and GLT1 [55], [56]. Also, a physical association and functional coupling between the α2 Na,K pump and glutamate transporters has been demonstrated [56].

We therefore hypothesize that CSD facilitation in the FHM2 mouse model is sustained by inefficient glutamate clearance by astrocytes and consequent enhanced cortical excitatory neurotransmission, particularly the NMDA receptor-mediated transmission during high-frequency action potential trains [57]. This glutamatergic hypothesis finds suggestive echoes in the recent report by Anttila et al. [58], where MTDH, a modulator of glutamate transporters has been associated to the common form of migraine with aura. In addition, a mutation of the glial excitatory aminoacid transporter type 1 (EAAT1) leads to neuronal hyperexcitability and subsequent seizures, hemiplegia, and episodic ataxia by impaired glutamate uptake [59]. While this scenario remains to be confirmed in the FHM2 mouse model, FHM1 models displayed an enhanced glutamatergic synaptic transmission due to increased Ca^2+^ influx through the mutant presynaptic CaV2.1 channels and increased probability of glutamate release at cortical pyramidal cell synapses [60]. A causative link between gain of function of glutamatergic transmission at recurrent cortical pyramidal cell synapses and facilitation of experimental CSD was demonstrated in the FHM1 mouse model [60]. Both FHM1 and FHM2 mice point to a model of CSD initiation, where the activation of NMDA receptors by glutamate released from recurrent cortical pyramidal cell synapses plays a key role in the positive feedback cycle that provokes CSD [4].

Furthermore, the absence of the α2 Na,K pump from the glial processes surrounding GABAergic terminals [55] suggests that FHM2 mutations fail to affect inhibitory neurotransmission, similarly to the FHM1 model, which showed unaltered inhibitory neurotransmission at synapses between fast-spiking interneurons and pyramidal cells [60].

We therefore propose that episodic disruptions of the excitation-inhibition balance and hyperactivity of cortical circuits due to excessive recurrent excitation underlie the vulnerability to “spontaneous” CSD ignition in both the rare forms of FHM1 and FHM2 and, probably, at least a fraction of common migraine cases.

## Materials and Methods

### Antibodies

Commercially available rabbit polyclonal antibody directed against α2 Na,K-ATPase isoform (cat. AB9094. Millipore, Billerica, MA, USA); mouse monoclonal antibodies for Na,K-ATPase alpha 1 isoform (α6F; Developmental Studies Hybridoma Bank, Iowa City, IA, USA), for Na,K-ATPase alpha 3 isoform (cat. MA3-915, Affinity Bio Reagents Suite 600 Golden, CO, USA), anti-bovine α-tubulin, mouse monoclonal antibody (cat. A11126, Molecular probes, Inc. 29851 Willow Creek Road, Eugene, OR, USA); GAPDH (6C5) mouse monoclonal antibody (sc-32233, Santa Cruz Biotechnology Inc., California, USA); Ubiquitin (P4D1), mouse monoclonal antibody (sc-8017, Santa Cruz Biotechnology Inc. CA, USA). ECL anti-mouse and anti-rabbit IgG and horseradish peroxidase (HRP)-linked species-specific whole antibodies were purchased from GE Healthcare. Polyclonal rabbit anti-goat IgG/HRP was obtained from Dako (Glostrup, Denmark). For immunofluorescence experiments, the following antibodies were used: monoclonal anti c-Myc (9E10) and rabbit anti- calnexin (Sigma-Aldrich, Milan, Italy). Secondary antibodies were conjugated with Alexa 488 and Alexa 596 (Invitrogen, Carlsbad, CA, USA).

### Generation of R887 knock-in mice

Procedures involving animals and their care were conducted in conformity with guidelines of the Institutional Animal Care and Use Committee at San Raffaele Hospital (Milan, Italy) in compliance with national (D.L. No. 116, G.U. Suppl. 40. Feb 18, 1992, Circolare No. 8 G.U., 14 Lug. 1994) and international (EEC Council Directive 86/609, OJ L 358, 1 DEC.12, 1987; National Institutes of Health Guide for the Care and Use of Laboratory Animals, U.S. National Research Council, 1996) laws and policies. Animals were housed in Specific Pathogen Free (SPF) conditions, maintained on a 12-h light/dark cycle, with free access to food and water. *Atp1a2* ^+/R887-neo^ knock-in mice were generated using homologous recombination in embryonic stem (ES) cells to modify the *Atp1a2* gene such that the exon 19 contained the human FHM-2 W887R mutation. In the targeting vector, the original TGG triplet codon (POSITION 2763, CODON 921) was changed into CGG by mutagenesis, creating the W887R mutation. Downstream of exon 19, a PGK-driven neo cassette flanked by LoxP sites was present. ES cells were electroporated, and clones were selected for homologous recombination by Southern blot analysis. The presence of the W887R mutation was tested by PCR using primers 5′-GGCTTCTTTACCTACTTTGTGATA-3′ and 5′-ATGCCCTGCTGGAACACTGAGTTG-3′ and subsequent sequencing analysis of exon 19. Targeted ES cells were injected into C57Bl/6J blastocysts and these transferred into pseudopregnant CD1 females to create chimeric animals. Chimeras were backcrossed with wild-type C57Bl/6J to generate F1 progeny and the agouti offsprings were genotyped for transmission of the mutant allele, generating transgenic line *Atp1a2* ^+/R887-neo^ knock-in mice. Heterozygous *Atp1a2* ^+/R887-neo^ knock-in mice were bred with transgenic mice expressing FLPe recombinase under the control of the human ACTB promoter (TgN(ACTFLPe)9205Dym; The Jackson Laboratory) to remove the neo cassette. Expression of FLPe recombinase as early as embryonic day 10.5 causes the Flippase recognition target (FRT) sites recombination and the removal of the *neo* cassette. Germ line transmission was obtained and transgenic line *Atp1a2* ^+/R887-neo^ was established. Mice were further bred with C57Bl/6J for seven generations, at this stage the background would nevertheless be >90% congenic. Heterozygous *Atp1a2* ^+/R88^ and *Atp1a2* ^+/+^ littermates were used for further analysis.

### Behavioral analysis

Sensory-motor function of mutant mice compared with controls was assessed by a modified version of the SHIRPA protocol primary screening [40]. Briefly, undisturbed behavior of each animal was first observed in its own home cage: body position, spontaneous activity and respiration rate were recorded, assigning a score to each behavior. In addition, manifestations of tremors, bizarre behaviors, stereotypes or convulsions were checked at this stage of the protocol. Thereafter mice were transferred individually to a new arena and were tested for transfer arousal, palpebral closing, piloerection, gait, pelvic and tail elevation, touch escape and positional passivity. There followed a sequence of manipulations using tail suspension and a grid across the width of the arena; animals were scored for trunk curl, limb grasping and grip strength. To complete the assessment, the animals were restrained in a supine position to record autonomic behaviors (heart rate, skin color, limb and abdominal tone, lacrimation, salivation) prior to measurement of the righting reflex after flip of the animal. Vocalizations and irritability (during supine restrain) were also recorded. Fear was assessed based on reaction to transfer to a new environment. A score was assigned to each behavioral test as described in Table 1.

### RT-PCR

Total RNA was extracted from embryonic mice (E19.5) (n = 9, 3 embryos for each genotype) neuronal (brain) tissues by Trizol method (Invitrogen, Carlsbad, CA, USA). RNA was reverse transcribed using random hexamers SuperScript® First-Strand Synthesis System (Invitrogen, Carlsbad, CA, USA) according to the manufacturer's instructions. *Atp1a2* cDNA was amplified using forward primer on exon 19 (5′-GGCTTCTTTACCTACTTTGTGATA-3′) and reverse primer on exon 20 (5′-ATGCCCTGCTGGAACACTGAGTTG-3′) with Hot Master Taq DNA polymerase (Eppendorf, Hamburg, Germany) at 94°C for 2 min, 35 cycles at 94°C for 30 s, 58°C for 30 s, 65°C for 30 s, and 65°C for 5 min. This strategy allows amplification of both endogenous wild-type and mutant allele (PCR product: 254 bp). The relative *Atp1a2* amount was normalized to the β-actin expression levels (610 bp PCR product). Since the R887 missense mutation introduces a new restriction site for MspI enzyme, the PCR product was subsequently digested with MspI (New England Biolabs, Ipswich, MA, USA) to discriminate the endogenous gene (uncut, band size: 254 bp band) and the mutant (cut, bands size: 178 bp+76 bp). PCR products were run on a 2% agarose gel in TAE buffer.

### Western blot analysis

To prevent proteolysis during the procedure, all steps were carried out on ice, and all buffers contained protease inhibitor cocktail (Roche, Mannheim, Germany) and phenylmethanesulfonyl fluoride (1 mM). Embryonic brains of the various genotypes (n = 12, 4 for each genotype) were processed simultaneously. For the extraction of membrane proteins, whole brain was homogenized with a glass-Teflon homogenizer in Sucrose solution (0.32 M Sucrose, 5 mM Hepes pH 7.4, 2 mM EDTA). After a short centrifugation (5000 rpm, 20′4°C) the supernatant was centrifuged for 1 hr at 42,000 rpm 1 h 4°C (Beckman, ultraTL100, rotor TL100.3) and the pellet resuspended in Sucrose buffer. Protein concentration was measured using the Bio-Rad Protein Assay according to the manufacturer's instructions. The preparation of cells and tissues (total brain, cortex and cerebellum) total lysates were performed adding RIPA buffer (50 mM Tris-HCl, pH 7.4, 150 mM NaCl, 0.5% sodium deoxycholate, 0.1% SDS, 2 mM EDTA, and 1% Igepal CA-630) to the collected samples and left 30′ on ice than the lysates were centrifuged 13000 g 20′4°C. The protein content of the supernatant was measured using the BCA protein assay with bovine serum albumin as standard. We resuspended equal amounts of proteins (15 µg each sample in 20 µl) in SDS-PAGE buffer (100 mM Tris-Glycine pH 6.8, 0.56 M mercaptoethanol, 2% SDS, 15% glycerol, and 0.1% BFB), and separated them for 2 h at 100volt in 8% SDS-polyacrilamide gels. Proteins were electrophoretically transferred to hybond ECL nitrocellulose membranes (GE Healthcare, Munich, Germany) and blots were blocked overnight with 5% non-fat milk 0.1% Tween-20 in PBS. The blocked blots were incubated for 2 h with subunit specific antibodies, washed three times for 10 minutes each with 0.1% Tween-20 in PBS then incubated with the appropriate peroxidase conjugated secondary antibodies. After another series of washes (three times for 10 minutes each) peroxidase was detected using a chemiluminescent substrate (GE Healthcare, Munich, Germany).

### Transfections and immunocytochemistry

Plasmid constructs were the same as in [17]) by metafectene (Biontex, Martinsried/Planegg, Germany) according to the manufacturer's instructions. We selected the ratio range of Metafectene (µl) to plasmids DNA (µg) of 5∶1. 48 h after transfection, we fixed HeLa cells in 4% paraformaldehyde (PFA) for 30 min at RT and blocked and permeabilized with 10% donkey serum 0.2% Triton-X100 in phosphate-buffered saline solution (PBS) for 30 min at RT. Permeabilized cells were then incubated with primary antibodies for 2 hr at RT, than washed (three times) in PBS, incubated with appropriate secondary antibodies and washed three times with PBS solution. We placed cells in fluorescent mounting medium (Dako Cytomation, Glostrup, Denmark) over microscope slides and confocal microscopy was performed on the Perkin Elmer UltraVIEW.

### Colocalization analysis

Immunofluorescence colocalization was visualized by confocal microscopy and analyzed by Wright Cell Imaging Facility (WCIF) colocalization plug-in of Image J software (http://www.uhnresearch.ca/facilities/wcif/imagej/colour_analysis.htm). The following parameters were measured: Pearson's correlation coefficient (Rr; 1, perfect correlation, to −1, perfect exclusion); Mander's overlap coefficient (R; 1, highest, to 0, random correlation); Ch1∶Ch2, the red∶green pixel ratio.

### Proteasome inhibitor

Proteasome inhibitor MG132 (carbobenzoxy-L-leucyl-L-leucyl-L-leucinal) was obtained from Sigma-Aldrich, Milan, Italy (cat. C2211). MG132 were dissolved in DMSO and applied to cells at the concentration of 10 µM, after 48 hours of transfection for the time periods indicated in the text and. An equivalent volume of DMSO was added to control cells. Anti ubiquitin antibody was used to reveal the increase of ubiquitinated proteins after proteasome inhibition.

### Cortical spreading depression

CSD was recorded as described in Van den Maagdenberg, et al. [46]. Briefly, mice (20–30 g) were anaesthetized with urethane (20% in saline; 6 ml/kg i.p.). Animals, mounted on a stereotaxic apparatus were continuously monitored for adequate level of anesthesia, temperature, heart rate and nociceptive reflexes. Blood oxygen saturation and flux as well as heart and breathing rates were monitored non-invasively using an oximeter (Starr, Life Science Corp.). Oxygen was supplied to maintain blood oxygenation above 93% for the entire duration of the experiment. Heart rate was between 400–600 beats/min, and breathing rate approximately 200 breaths/min. Animals not meeting these criteria were excluded from our sample. To record CSD three holes were drilled in the skull over the left hemisphere. The first corresponded to the occipital cortex and was used for access of the electrical stimulation electrode (0 mm A-P, 2 mm M-L from lambda). The second hole, at the parietal cortex (1 mm M-L, 1 mm caudal to bregma) and the third hole, at the frontal cortex (1 mm M-L, 1 mm rostral to bregma), were used for placement of the CSD recording electrodes. The steady (DC) potential was recorded with glass micropipettes filled with NaCl (3 M, tip resistance 1–2 MΩ) inserted 200 µm below the dural surface. An Ag/AgCl reference electrode was placed subcutaneous above the nose. Cortical stimulation was conducted using a copper bipolar electrode (0.2 mm tip diameter, 0.3 mm intertip distance) placed on the cortex surface after removing the dura. Single pulses of increasing intensity (20, 30, 40, 50, 60, 80, 100, 120, 140, 160, 180, 200, 230, 260, 290, 320, 350, 380, 430, 480, 530, 600, 700, 800, 900, 1000 µA) were applied for 100 ms at 3-min intervals by using a stimulus isolator/constant current unit (WPI, USA) until a CSD event was observed [13]. DC cortical potential was amplified (10×) and low-pass filtered at 200 Hz (Cyberamp, Axon Instruments, Union City, CA). Signals were continuously digitized and recorded using Labview data acquisition and analysis system. The minimal stimulus intensity at which a CSD event was elicited was taken as the CSD threshold. In all mice, when CSD was elicited, recordings were continued for 90 min to detect multiple CSDs. To estimate CSD propagation velocity, the distance between the two recording electrodes was divided by the time elapsed between the CSD onsets at the first and second recording sites. The percentage of mice with multiple CSD events was determined only from the mice that could be recorded for one full 90 min following the first detected event. CSD duration was measured at half-maximal amplitude [13]. Because no difference in CSD threshold and propagation rate was observed between male (N = 11 wild type and N = 11 mutants) and female (N = 7 wild type and N = 9 mutants) within each genotype (wild type: threshold male 20.7±2.1 µC, female 18.7±3.5 Mann-Whitney test p = 0.61; propagation rate male 3.9±0.42 mm/min, female 3.8±0.66 mm/min Mann-Whitney test p = 0.86; mutants: threshold male 13.4±3.0 µC, female 12.6±1.3 t-test p = 0.82; propagation rate male 5.2±0.32 mm/min, female 5.6±0.84 mm/min Mann-Whitney test p = 0.94) the results from males and females were pooled.

### Statistical analysis

For SHIRPA protocol primary screening, comparisons were performed with the Mann-Whitney nonparametric test. The Student's *t*-test with one-tail distribution was used for significance calculation in densitometric analysis.

Statistical analysis for CSD recordings was performed using Sigma Stat 3.1 (Systat Software, Chicago IL USA). Multiple groups were compared by ANOVA followed by post-hoc comparisons applying Bonferroni correction or Holm-Sidak test. When two groups were compared, t-test was applied. Normality and homoschedasticity of the data was checked. Data not normally distributed were compared using nonparametric Kruskal-Wallis ANOVA or Mann-Whitney rank sum test. Significance level was equal to 0.05. Data are reported as average ± SEM.

## References

[1] Russell MB, Iversen HK, Olesen J (1994). Improved description of the migraine aura by a diagnostic aura diary.. Cephalalgia.

[2] Goadsby PJ, Lipton RB, Ferrari MD (2002). Migraine–current understanding and treatment.. N Engl J Med.

[3] Pietrobon D, Striessnig J (2003). Neurobiology of migraine.. Nat Rev Neurosci.

[4] Pietrobon D (2005). Migraine: new molecular mechanisms.. Neuroscientist.

[5] Lauritzen M (1994). Pathophysiology of the migraine aura. The spreading depression theory.. Brain.

[6] Cutrer FM, Sorensen AG, Weisskoff RM, Ostergaard L, Sanchez del Rio M (1998). Perfusion-weighted imaging defects during spontaneous migrainous aura.. Ann Neurol.

[7] Hadjikhani N, Sanchez Del Rio M, Wu O, Schwartz D, Bakker D (2001). Mechanisms of migraine aura revealed by functional MRI in human visual cortex.. Proceedings of the National Academy of Sciences of the United States of America.

[8] Bowyer SM, Aurora KS, Moran JE, Tepley N, Welch KM (2001). Magnetoencephalographic fields from patients with spontaneous and induced migraine aura.. Ann Neurol.

[9] Milner PM (1958). Note on a possible correspondence between the scotomas of migraine and spreading depression of Leao.. Electroencephalogr Clin Neurophysiol.

[10] Bolay H, Reuter U, Dunn AK, Huang Z, Boas DA (2002). Intrinsic brain activity triggers trigeminal meningeal afferents in a migraine model.. Nat Med.

[11] Dalkara T, Zervas NT, Moskowitz MA (2006). From spreading depression to the trigeminovascular system.. Neurol Sci.

[12] Zhang X, Levy D, Noseda R, Kainz V, Jakubowski M (2010). Activation of meningeal nociceptors by cortical spreading depression: implications for migraine with aura.. J Neurosci.

[13] Ayata C, Jin H, Kudo C, Dalkara T, Moskowitz MA (2006). Suppression of cortical spreading depression in migraine prophylaxis.. Ann Neurol.

[14] Wessman M, Kaunisto MA, Kallela M, Palotie A (2004). The molecular genetics of migraine.. Ann Med.

[15] Ferrari MD (2008). Migraine genetics: a fascinating journey towards improved migraine therapy.. Headache.

[16] Ophoff RA, Terwindt GM, Vergouwe MN, van Eijk R, Mohrenweiser H (1996). A 3-Mb region for the familial hemiplegic migraine locus on 19p13.1-p13.2: exclusion of PRKCSH as a candidate gene. Dutch Migraine Genetic Research Group.. Eur J Hum Genet.

[17] De Fusco M, Marconi R, Silvestri L, Atorino L, Rampoldi L (2003). Haploinsufficiency of ATP1A2 encoding the Na+/K+ pump alpha2 subunit associated with familial hemiplegic migraine type 2.. Nat Genet.

[18] Dichgans M, Freilinger T, Eckstein G, Babini E, Lorenz-Depiereux B (2005). Mutation in the neuronal voltage-gated sodium channel SCN1A in familial hemiplegic migraine.. Lancet.

[19] Riant F, De Fusco M, Aridon P, Ducros A, Ploton C (2005). ATP1A2 mutations in 11 families with familial hemiplegic migraine.. Hum Mutat.

[20] Jurkat-Rott K, Freilinger T, Dreier JP, Herzog J, Gobel H (2004). Variability of familial hemiplegic migraine with novel A1A2 Na+/K+-ATPase variants.. Neurology.

[21] Kaunisto MA, Harno H, Vanmolkot KR, Gargus JJ, Sun G (2004). A novel missense ATP1A2 mutation in a Finnish family with familial hemiplegic migraine type 2.. Neurogenetics.

[22] Pierelli F, Grieco GS, Pauri F, Pirro C, Fiermonte G (2006). A novel ATP1A2 mutation in a family with FHM type II.. Cephalalgia.

[23] Spadaro M, Ursu S, Lehmann-Horn F, Veneziano L, Antonini G (2004). A G301R Na+/K+ -ATPase mutation causes familial hemiplegic migraine type 2 with cerebellar signs.. Neurogenetics.

[24] Vanmolkot KR, Kors EE, Hottenga JJ, Terwindt GM, Haan J (2003). Novel mutations in the Na+, K+-ATPase pump gene ATP1A2 associated with familial hemiplegic migraine and benign familial infantile convulsions.. Annals of Neurology.

[25] Deprez L, Weckhuysen S, Peeters K, Deconinck T, Claeys KG (2008). Epilepsy as part of the phenotype associated with ATP1A2 mutations.. Epilepsia.

[26] Vanmolkot KR, Stroink H, Koenderink JB, Kors EE, van den Heuvel JJ (2006). Severe episodic neurological deficits and permanent mental retardation in a child with a novel FHM2 ATP1A2 mutation.. Ann Neurol.

[27] Ambrosini A, D'Onofrio M, Grieco GS, Di Mambro A, Montagna G (2005). Familial basilar migraine associated with a new mutation in the ATP1A2 gene.. Neurology.

[28] Todt U, Dichgans M, Jurkat-Rott K, Heinze A, Zifarelli G (2005). Rare missense variants in ATP1A2 in families with clustering of common forms of migraine.. Hum Mutat.

[29] Blanco G, Mercer RW (1998). Isozymes of the Na-K-ATPase: heterogeneity in structure, diversity in function.. Am J Physiol.

[30] Hu YK, Kaplan JH (2000). Site-directed chemical labeling of extracellular loops in a membrane protein. The topology of the Na,K-ATPase alpha-subunit.. J Biol Chem.

[31] Crambert G, Hasler U, Beggah AT, Yu C, Modyanov NN (2000). Transport and pharmacological properties of nine different human Na, K-ATPase isozymes.. J Biol Chem.

[32] de Carvalho Aguiar P, Sweadner KJ, Penniston JT, Zaremba J, Liu L (2004). Mutations in the Na+/K+ -ATPase alpha3 gene ATP1A3 are associated with rapid-onset dystonia parkinsonism.. Neuron.

[33] McGrail KM, Phillips JM, Sweadner KJ (1991). Immunofluorescent localization of three Na,K-ATPase isozymes in the rat central nervous system: both neurons and glia can express more than one Na,K-ATPase.. J Neurosci.

[34] Pietrobon D (2007). Familial hemiplegic migraine.. Neurotherapeutics.

[35] Tavraz NN, Friedrich T, Durr KL, Koenderink JB, Bamberg E (2008). Diverse functional consequences of mutations in the Na+/K+-ATPase alpha2-subunit causing familial hemiplegic migraine type 2.. J Biol Chem.

[36] Tavraz NN, Durr KL, Koenderink JB, Freilinger T, Bamberg E (2009). Impaired plasma membrane targeting or protein stability by certain ATP1A2 mutations identified in sporadic or familial hemiplegic migraine.. Channels (Austin).

[37] Jorgensen PL, Hakansson KO, Karlish SJ (2003). Structure and mechanism of Na,K-ATPase: functional sites and their interactions.. Annu Rev Physiol.

[38] Koenderink JB, Zifarelli G, Qiu LY, Schwarz W, De Pont JJ (2005). Na,K-ATPase mutations in familial hemiplegic migraine lead to functional inactivation.. Biochim Biophys Acta.

[39] James PF, Grupp IL, Grupp G, Woo AL, Askew GR (1999). Identification of a specific role for the Na,K-ATPase alpha 2 isoform as a regulator of calcium in the heart.. Mol Cell.

[40] Rogers DC, Fisher EM, Brown SD, Peters J, Hunter AJ (1997). Behavioral and functional analysis of mouse phenotype: SHIRPA, a proposed protocol for comprehensive phenotype assessment.. Mamm Genome.

[41] van den Maagdenberg AM, Pizzorusso T, Kaja S, Terpolilli N, Shapovalova M (2010). High cortical spreading depression susceptibility and migraine-associated symptoms in Ca(v)2.1 S218L mice.. Ann Neurol.

[42] Ikeda K, Onaka T, Yamakado M, Nakai J, Ishikawa TO (2003). Degeneration of the amygdala/piriform cortex and enhanced fear/anxiety behaviors in sodium pump alpha2 subunit (Atp1a2)-deficient mice.. J Neurosci.

[43] Moseley AE, Lieske SP, Wetzel RK, James PF, He S (2003). The Na,K-ATPase alpha 2 isoform is expressed in neurons, and its absence disrupts neuronal activity in newborn mice.. J Biol Chem.

[44] Onimaru H, Homma I (2007). Spontaneous oscillatory burst activity in the piriform-amygdala region and its relation to in vitro respiratory activity in newborn rats.. Neuroscience.

[45] Moseley AE, Williams MT, Schaefer TL, Bohanan CS, Neumann JC (2007). Deficiency in Na,K-ATPase alpha isoform genes alters spatial learning, motor activity, and anxiety in mice.. J Neurosci.

[46] van den Maagdenberg AM, Pietrobon D, Pizzorusso T, Kaja S, Broos LA (2004). A Cacna1a knockin migraine mouse model with increased susceptibility to cortical spreading depression.. Neuron.

[47] Eikermann-Haerter K, Dilekoz E, Kudo C, Savitz SI, Waeber C (2009). Genetic and hormonal factors modulate spreading depression and transient hemiparesis in mouse models of familial hemiplegic migraine type 1.. J Clin Invest.

[48] Moskowitz MA, Bolay H, Dalkara T (2004). Deciphering migraine mechanisms: clues from familial hemiplegic migraine genotypes.. Ann Neurol.

[49] D'Ambrosio R, Gordon DS, Winn HR (2002). Differential role of KIR channel and Na(+)/K(+)-pump in the regulation of extracellular K(+) in rat hippocampus.. J Neurophysiol.

[50] Ransom CB, Ransom BR, Sontheimer H (2000). Activity-dependent extracellular K+ accumulation in rat optic nerve: the role of glial and axonal Na+ pumps.. J Physiol.

[51] Somjen GG (2001). Mechanisms of spreading depression and hypoxic spreading depression-like depolarization.. Physiol Rev.

[52] Haglund MM, Schwartzkroin PA (1990). Role of Na-K pump potassium regulation and IPSPs in seizures and spreading depression in immature rabbit hippocampal slices.. J Neurophysiol.

[53] Clapcote SJ, Duffy S, Xie G, Kirshenbaum G, Bechard AR (2009). Mutation I810N in the alpha3 isoform of Na+,K+-ATPase causes impairments in the sodium pump and hyperexcitability in the CNS.. Proc Natl Acad Sci U S A.

[54] Pellerin L, Magistretti PJ (1997). Glutamate uptake stimulates Na+,K+-ATPase activity in astrocytes via activation of a distinct subunit highly sensitive to ouabain.. J Neurochem.

[55] Cholet N, Pellerin L, Magistretti PJ, Hamel E (2002). Similar perisynaptic glial localization for the Na+,K+-ATPase alpha 2 subunit and the glutamate transporters GLAST and GLT-1 in the rat somatosensory cortex.. Cereb Cortex.

[56] Rose EM, Koo JC, Antflick JE, Ahmed SM, Angers S (2009). Glutamate transporter coupling to Na,K-ATPase.. J Neurosci.

[57] Tzingounis AV, Wadiche JI (2007). Glutamate transporters: confining runaway excitation by shaping synaptic transmission.. Nat Rev Neurosci.

[58] Anttila V, Stefansson H, Kallela M, Todt U, Terwindt GM Genome-wide association study of migraine implicates a common susceptibility variant on 8q22.1.. Nat Genet.

[59] Jen JC, Wan J, Palos TP, Howard BD, Baloh RW (2005). Mutation in the glutamate transporter EAAT1 causes episodic ataxia, hemiplegia, and seizures.. Neurology.

[60] Tottene A, Conti R, Fabbro A, Vecchia D, Shapovalova M (2009). Enhanced excitatory transmission at cortical synapses as the basis for facilitated spreading depression in Ca(v)2.1 knockin migraine mice.. Neuron.

